# Needs Analysis About Intercultural Communicative Competence Among Undergraduate Tourism Students

**DOI:** 10.1007/s10936-023-10012-1

**Published:** 2023-09-12

**Authors:** Tran Thi Thu Trang, Vo Tu Phuong

**Affiliations:** 1 University of Foreign Languages and International Studies, Hue University, 57 Nguyen Khoa Chiem St, Hue, Vietnam; 2 Khanh Hoa University, 1 Nguyen Chanh St, Nha Trang, Vietnam

**Keywords:** Needs analysis, Intercultural communication competence, English for tourism purposes, Tourism majored undergraduate

## Abstract

With both the quantitative and qualitative data from 628 responses to a set of questionnaire collected from the undergraduates of three educational institutions in central Vietnam, this study analyzed learners’ needs of ***intercultural communication competence*** (ICC) related to their studying of English for tourism purposes and future occupations. The methodology used for data analysis including semi-structured interviews, and the questionnaire. The findings showed that the students preferred intercultural language learning activities referring to authentic materials and real-life experience. The results also revealed the participants’ great needs of various ICC attitudes and regular tasks in tourism workplaces. Particularly, they had positive attitudes in intercultural communication, and higher needs of tasks for improving discourse and behavioural competences more than other ICC dimensions. The study has implications for tourism learners, educators and related stakeholders to raise their awareness in learning, teaching and developing this long-lasting competence.

## Introduction

***Needs analysis*** (NA) is crucial for depicting learners’ profiles to examine and prioritize the needs of competences that students must be equipped with (Richards et al., 1992) in English language learning generally (Finney, 2002; Richards, 2021) and English for specific purposes particularly (Dudley-Evans & St. John, 1998; Prachanant, 2012). In the realm of teaching and learning ***English for tourism purposes*(ETP)**, scholars are generating considerable interest in terms of NA related to learners’ necessary proficiency (Bach, 2015; Ratminingsih et al., 2018). Along with language competence, senior students, the future labour, of tourism industry also need to acquire ***intercultural communication competence*(ICC)** to interact efficiently in intercultural contexts (Grobella, 2015; Koc, 2021). However, to the best of our knowledge, at tertiary level of Vietnamese context, there has never been such a research investigating the learners’ needs of this long-life competence, ICC, in their ETP courses and in an intercultural working environment they are engaging like tourism domain.

The current research seeks to address the students’ needs of ICC pertaining to their current language learning and their future jobs. Particularly, the present study aims to examine the undergraduates’ learning needs and target needs (Hutchinson & Walters, 1987) of ICC related to their ongoing ETP courses as well as their subsequent careers in the tourism field. While there has been considerable research on NA for language proficiency in ETP, there is a lack of studies examining learners’ needs for ICC in tertiary-level Vietnamese contexts. This paper aims at raising the learners’ awareness of the needs of a fundamental competence for tourism staff, and adjusting the ETP teachers’ method of intercultural language teaching. Additionally, the business owners and professional trainers at hotels and travel companies should consider to equip their staff with this crucial competence to provide quality services (Liu et al., 2022) and meet tourists’ need (Sharma, 2018). Since there have not been similar studies conducted in tourism domain in Vietnamese ***EFL settings*** (English as a foreign language setting), this research will be considered as a referent point for further researches referring to NA of ICC in resembling context and beyond.

## Literature Review

### Intercultural Communication Competence and Intercultural Communication Competence in Tourism

Intercultural communication competence is “*the individual’s ability to interact with people from another country and culture in a foreign language by acting as a mediator between people of different cultural origins*” (Byram, 1997, pp. 48–73). Intercultural communication competence refers to the ability to effectively and appropriately communicate with individuals from different cultural backgrounds. It involves understanding and respecting cultural differences, as well as possessing the necessary knowledge, skills, and attitudes to navigate and bridge cultural gaps. It is considered as the prerequisite of communicating effectively and appropriately between people coming from different cultures. ICC components and models have been discussed by many scholars (Balboni, 2006; Hoff, 2020). Some prominent authors and their works in this field include Milton J. Bennett (2017) is known for his Developmental Model of Intercultural Sensitivity (DMIS), which describes six stages of intercultural sensitivity. His work focuses on the psychological and cognitive aspects of intercultural communication competence. Janet M. Bennett (2004) Janet Bennett has contributed significantly to the field of intercultural communication competence. Her book “***Developing Intercultural Competence: A Reader***” is a comprehensive collection of essays that explore various dimensions of intercultural competence. Neuliep (2006) is the author of the book “***Intercultural Communication: A Contextual Approach***.“ He explores the theories and concepts related to intercultural communication, including intercultural competence and its relevance in different contexts. Ting-Toomey (1999) (2022) has written several books on intercultural communication, including “***Communicating Across Cultures***” and “Understanding Intercultural Communication.“ Her work focuses on the role of cultural identities, face-saving, and conflict resolution in intercultural interactions. Jandt (2021) has authored several textbooks on intercultural communication, including “***An Introduction to Intercultural Communication: Identities in a Global Community***.“ His works cover various aspects of intercultural communication, including intercultural competence, cultural identity, and conflict resolution.

Among these, Byram’s (1997) model of ICC is acknowledged as the cornerstone in the domain of foreign language teaching and learning with a clear position of both teachers and learners (Behrnda & Porzelt, 2012; Newton & Shearn, 2010; Sercu, 2005). Hence, Byram’s (1997) model of ICC is employed as the framework in this study for its explicit and comprehensive features as well as the suitability with the research purposes of examining ICC in foreign language education.

According to Byram (1997, 2021), ICC comprises linguistic competence, sociolinguistic competence, discourse competences and ***intercultural competence*** (IC). IC includes Knowledge (savoirs), Skills of interpreting and relating (savoir comprendre), Skills of discovery and interaction (savoir apprendre/faire), Critical cultural awareness (savoir s’engager), and Attitudes (savoir être). Byram (2006, pp. 22–26) summarized the five competences of IC into affective capacities/attitudes, behaviour and cognitive capacities. Particularly, the affective capacity refers to “*acknowledgement of the identities of others, respect for otherness, tolerance for ambiguity and empathy*”, meanwhile the behavioural capacity means “*behaviour, flexibility, communicative awareness*”, and cognitive capacity covers “*knowledge, knowledge discovery, interpreting and relating, and critical cultural awareness*” (Byram, 2006, pp. 22–26).

Ho (2009) and Chlopek (2008) highlight the significance of intercultural language learning in modern language education. By focusing on both linguistic competence and intercultural communicative competence, educators aim to equip language learners with the skills and knowledge necessary for successful intercultural communication in today’s globalized world.

Ho (2009) argued that in the field of education, there is a current emphasis on intercultural language learning in modern languages. The goal is to equip language learners with the necessary skills for effective intercultural communication. According to Chlopek (2008), to truly master a language, it is typically necessary to have some understanding of the culture associated with that language.

Intercultural language learning refers to the process of developing not only linguistic competence but also intercultural communicative competence (ICC). Linguistic competence involves acquiring the necessary language skills, such as vocabulary, grammar, and pronunciation, to communicate effectively in a foreign language. On the other hand, ICC refers to the ability to understand and navigate the cultural aspects of communication, including norms, values, and social conventions.

By incorporating intercultural language learning into education, learners are exposed to cultural elements that are integral to language use. This approach recognizes that language is not merely a system of words and grammar, but it is deeply intertwined with the culture of its speakers. Therefore, understanding the cultural context is crucial for successful communication and language learning.

Intercultural language learning allows learners to develop a broader perspective and appreciation for different cultures. It helps them become more aware of their own cultural biases and encourages them to be open-minded and respectful when interacting with speakers of other languages. By integrating cultural knowledge into language education, learners are better prepared to engage in meaningful and effective intercultural communication.

Krieger (2005) argues that in an English as a Foreign Language (EFL) class, learners often have limited exposure to international cultures because they typically live in their own country and are monolingual. This lack of direct contact with English-speaking environments outside the classroom can result in limited opportunities for learners to engage in authentic English communication (Chomchuen & Rattanasak 2018). As a consequence, their awareness of intercultural communicative competence (ICC) may be limited.

To address this issue and enhance learners’ ICC, Kongkerd (2013) suggests that teaching practices should be based on real-life situations within intercultural communities. By incorporating authentic materials, cultural references, and real-world scenarios into the curriculum, learners can gain a deeper understanding of how language and culture intersect in genuine contexts.

Moreover, Yu et al. (2001) recommend a model of intercultural competence for tour guide which is adapted for all tourism staff in this study. Yu et al. (2001) also include the same competences with Byram’s (2006) summarized IC capacities, i.e. *affective, behavioural* and *cognitive* competences. Particularly, according to Yu et al. (2001, pp. 80–81), *affective* competence refers to being sympathetic, open-minded and tactful when serving guests; meanwhile, *behavioural* dimension means “communication skills and interpersonal interaction”, and *cognitive* facet is defined as tourism employees’ understanding of their own and their customers’ cultures. Briefly, in this research, ICC of labour working in tourism industry, adapted from the models of Byram (2006) and Yu et al. (2001), covers linguistic, sociolinguistic, discourse competences as well as affective, behavioural and cognitive capacities.

### Teaching for Intercultural Communication Competence in Courses of English for Tourism Purposes

Teaching ETP is crucial for the tourism undergraduates’ later employment, as they are undertaking job duties with foreign managers, co-workers and guests coming from various national origins. This requires learners to speak English with people from different cultural backgrounds. In order to communicate efficiently in various cultural contexts, students and labour of this industry need awareness of both linguistic and non-linguistic factors to avoid barriers often caused by cultural misunderstanding (Kaur, 2016; Ho, 2020). Hence, in ETP courses, teachers not only improve learners’ language proficiency but also help them to enhance ICC (Liu et al., 2022).

Multiple intercultural activities have been recommended by many scholars (Reid, 2015; Hartmann & Ditfurth, 2007). For instance, Huber and Reynolds (2014) recommend seven intercultural language learning activities. These activities are utilized in this study thanks to their feasibility to observe and categorize multiple intercultural learning activities in both nonformal and formal intercultural educational settings. They include (1) activities emphasizing multiple perspectives, (2) role plays, simulations and drama, (3) theatre, poetry and creative writing, (4) ethnographic tasks, (5) use of films and texts, (6) image-making in class, and (7) use of social media and other online tools. Since these suggested activities are employed in diverse intercultural teaching and learning contexts (Echcharfy, 2019), Huber and Reynolds’ (2014) taxonomy of intercultural communicative activities was used in the present study to investigate the undergraduates’ ICC needs in ETP courses.

### Needs Analysis

Generally, the term needs analysis is defined as the activities employed in collecting information considered as the foundation for developing a language course to meet the needs of a specific group of learners (Richards, 2021; Richards et al., 1992).

The development of needs analysis approaches to ESP have been discussed by several scholars (Flowerdew, 2012; Huhta et al., 2013). According to Huhta et al. (2013), the development of needs analysis approaches can be classified into two groups. The first group refers to language-centered approaches which focused on functions, notions, and the four language skills. This group covers target situation analysis, present situation analysis, etc. (Robinson, 1991; West, 1997; Dudley-Evans & St. John, 1998). The second group is a comprehensive task-based approach, as seen in the work of Long (2005), whose needs analysis is primarily based on tasks. Huhta et al. (2013) also suggest a later approach, a holistic approach to ESP, which does not refer to a clear division between the two groups of needs analysis approaches.

In order to seek specific requirements for the current language learning and later professions, a needs analysis process examining both learners’ learning and target situations should be involved (Ratmingsih et al., 2018; Richterich, J. Chancerel, 1980). Particularly, the needs analysis approach suggested by Long (2005) is entirely satisfactory with the purpose of examining the target needs in this study since it is based on the real-world tasks, and it aims to meet the learners’ “*diverse psycholinguistic and communicative needs*” (Long, 2015, p. 14).

#### Needs Related to Future Jobs

As we look toward the future, several emerging trends and needs can be observed in relation to future jobs. Here are some prominent perspectives on this topic: **The WEF’s Future of Jobs Report** (2020) emphasizes the importance of skills such as complex problem-solving, critical thinking, creativity, and emotional intelligence while **McKinsey’s research**, a global management consulting firm, conducts extensive research on various topics, including the future of work and skills, emphasizes the growing demand for skills in advanced technological areas, including data analytics, machine learning, and programming. They also stress the significance of adaptability, entrepreneurship, and a continuous learning mindset to thrive in a rapidly changing job market and **Deloitte’s analysis**, a multinational professional services firm, has also conducted research and analysis on the importance of digital skills in the workforce. emphasizes the need for digital skills, particularly in areas like cybersecurity, data science, and AI. They also highlight the importance of developing uniquely human skills such as creativity, critical thinking, and leadership, as automation takes over routine tasks.

Here are some key areas that are expected to be in high demand: Technological literacy, Adaptability and continuous learning, Critical thinking and problem-solving, Emotional intelligence and interpersonal skills, Sustainability and environmental awareness, Data literacy and analysis, and Cross-cultural competence, and etc. It’s important to note that the job market is constantly evolving, and new needs may emerge over time. Developing a diverse skill set, staying updated with industry trends, and cultivating a willingness to learn and adapt will be key to thriving in future job markets.

#### Attitudes

Attitudes play a significant role in the workplace and can greatly influence an individual’s success and satisfaction in their career. Fred Luthans (2011) has a book “Organizational Behavior: An Evidence-Based Approach, “provide valuable insights into the role of attitudes in the workplace. Here are some attitudes that can be beneficial for future jobs: growth mindset, open-mindedness, proactiveness, resilience, adaptability, collaboration and teamwork, professionalism and integrity, curiosity and continuous learning and etc. Developing and embodying these positive attitudes can help individuals thrive in future jobs, adapt to changing circumstances, and contribute effectively in dynamic work environments.

#### Perceptions

Perceptions, or the way individuals interpret and make sense of the world around them, can have a significant impact on various aspects of work and career. Albert Bandura, a renowned psychologist, has written several influential books that delve into his social cognitive theory and the role of perceptions in shaping behavior and motivation. Here are some of his notable books: “Social Learning Theory” (Bandura, 1977): In this book, Bandura introduces his social learning theory, which emphasizes the importance of observational learning, modeling, and cognitive processes in understanding human behavior. Another book is “Self-Efficacy: The Exercise of Control” (Bandura, 1997). This book focuses specifically on the concept of self-efficacy, which refers to an individual’s belief in their own capabilities to succeed in specific situations or accomplish particular tasks. So there ara perceptions that can influence success in future jobs: positive outlook, self-efficacy, embracing diversity, change readiness, customer-centricity, ethical awareness, continuous learning etc. It’s important to note that perceptions can vary among individuals and may be influenced by personal experiences, beliefs, and values. Cultivating positive perceptions and adopting a growth-oriented mindset can empower individuals to navigate the complexities of future jobs, embrace opportunities for growth, and achieve long-term success.

### Needs Analysis of ICC in ETP

In the teaching and learning ETP realm, scholars claim that students, also the future tourism staff, need to be equipped both language proficiency and ICC (Beydilli & Kurt, 2020; Liu et al., 2022). ICC is a crucial competence for tourism personnel engaging in continuous and intense communication with their guests, colleagues and managers coming from diverse cultural backgrounds (Gibson & Zhong, 2005; Koc, 2017; Lieberman & Gamst, 2015). Hence, NA of ICC should be involved for learners to raise their awareness in learning this long-lasting competence as well as for ETP educators’ and related stakeholders to reconsider how to equip their students and employees with ICC for their jobs. However, as far as we know, there are few researches investigating learners’ NA of English language competence (Bach, 2015; Do and Nguyen, 2021) but studies about NA of ICC in Vietnamese tertiary context, especially in tourism field, are unexplored.

Needs analysis in this study focuses on clarifying both learning needs and target needs of students in learning ETP. To specify, the learning needs refer to the ICC needs related to the students’ current intercultural learning in ETP courses; meanwhile, the learners’ needs of ICC performed in their future workplaces belong to target needs. Clearly, there is a close link between the learner’s needs in language learning process and their upcoming careers. Each kind of need shares an important role in determining the learners’ success in the learning process and their upcoming professions. In addition, after examining needs analysis, by identifying elements of students’ current and target ICC needs, and using them as the basic of ETP instruction, teachers will be able to provide students with the specific language and competence they need to succeed in their future careers (Dou et al., 2023).

### Previous Studies

In the stance of needs analysis of ETP in general, many researchers studied the needs analysis of language skills in ETP courses. The studies of Alhumaidan and Alghamdi (2023) from Saudi Arabia, Kusumastiti and Palupiningsih (2021) from Indonesia, Uysal et al. (2018) from Turkey and Prachanant (2012) from Thailand all searched for the English language needs analysis of hotel students and related stakeholders, students only or tourism employees. The findings of these studies revealed that there were some special English language needs and interests for the tourism students and employees. For instance, the most needed English language skill was speaking (Alhumaidan & Alghamdi, 2023), listening and speaking (Uysal et al., 2018), or reading (Kusumastiti & Palupiningsih, 2021). Meanwhile, Prachanant’s (2012) findings showed that speaking skill was most important; then came listening, reading and writing skills. The tourism staff’s common problems when using English included incapacity to understand foreign tourists’ accents, unsuitability of language use, insufficient vocabulary, and inadequacy of grammar knowledge.

More particularly focused on investigating ICC in the domain of ESP, the study with a combination of quantitative and qualitative methods to examine the ICC of 120 office personnel working in business corporates in Bangkok of Inkaew (2022) exposed that business workers was equipped with a certain degree of ICC; however, at a low to moderate level. These employees perceived that training program related to ICC in general and IC awareness in particular brought them many benefits for their professional and personal lives. In tourism field, Fujita et al. (2017) carried out a mixed method study to examine the communication needs of people at two Japanese rural destinations. The results showed that local people involved in tourism domain generally had positive attitudes towards foreign tourists. However, they needed to improve their language proficiency and communicative skills to interact efficiently with international visitors.

In Vietnamese ELT context, Do and Nguyen (2021) investigated the needs for English learning of 180 English-majored students participating in a questionnaire-interview survey. The findings indicated that the two skills they needed most were speaking and listening. However, the classroom practices which did not focus on these skills were unsatisfactory from the learners’ viewpoint. Also in Vietnamese setting, the quantitative approach in Bach’s (2015) study aimed to examine needs and problems of English language use of tourism employees at international travel companies in Hue, Vietnam. Through the findings, speaking skill appeared to be the most important need among English language skills of tourism employees in their workplaces, following by listening, writing and reading respectively. Whereas, reading was the most faced problem of English competence at employees’ routine jobs rather than writing, listening and speaking skills.

Succinctly, the related studies have shown that needs analysis are vital in ELT and ETP education. The majority of the researches reviewed so far have investigated mainly the tourism learners’ or employees’ needs of English language competence. There were few studies examined needs of ICC in ESP (Fujita et al., 2017; Inkaew, 2022), and researches focus on this issue in tourism domain at tertiary level of Vietnamese context are still underexplored.

## Methodology

### Research Design

To examine the ICC needs analysis (NA) of the tourism-majored students, the theory of ICC by Byram (2006), the theory of intercultural competence (IC) for tourism majors by Yu et al. (2001), and Long’s (2005) framework of task-based NA approach were utilized to develop this research’s questionnaire.

According to Long (2015), if a research starts, or even worse, starts and finishes with a questionnaire, it “*runs the risk of precluding discovery of relevant information, as survey items are effectively tests of the survey designer’s preconceptions about the needs in question*” (p. 141). Therefore, the questionnaire used in this study was not built based on the “*preconceptions*” of the researcher about the students’ needs of ICC, but it was built on the students’ needs of intercultural language learning and ICC real-life tasks set up after the interview with experts in tourism domain.

This study has three phases. Firstly, semi-structured interviews were conducted with a random sample of ten senior students enrolled in ETP courses in the second semester of the academic year 2021–2022 and ten volunteered experts of tourism majors. Secondly, the interviews were summarized for specific information that described these groups of students’ and professionals’ needs in order to develop the questionnaire to investigate the undergraduates’ ICC needs in ETP courses and the needs in their future workplaces. Finally, the questionnaire, which was piloted with a group of 20 seniors chosen randomly and had slight changes in wording to enhance clarity, were administered to the entire sample of students.

### Participants

There were about 1,000 students enrolled in ETP classes in three research sites of this study in the school year 2021–2022. If the confidence level is 95%; the margin of error is 5%; the population proportion is 90%; the population size is 1,000; the sample size needed is 122 (Maple Tech., n.d.). In this study, a total of 628 students, from a junior college and two universities, participated in the questionnaire survey of this study. There were 116 students from the college and 512 students from the two universities. They were the accessible sample and chosen randomly. Three quarters of them were female (75.5%), and only 158 were male (24.5%). The undergraduates, who were mostly 21 to 22 years old and had learned English about eight to twelve years with their self-assessment of English proficiency at intermediate level mainly, hoped to work in the fields of Hotels and Restaurants, Event and Tour Operation as well as Tourism Management in their future jobs.

### Instrument

This study employed a set of questionnaire including eight questions with both closed-ended and open-ended parts (Table 1).

**Table 1:** Summary of The Undergraduates’ Questionnaire

Section	Questions	Types of question	Content
Part A: General information	Q1 – Q5	Closed-ended	Demographic characteristics
Part B: Needs related to ICC in ETP courses	Q6	Q6.1 – Q6.7 Closed-ended Q6.8 Open-ended	Kinds of preferred intercultural language learning activities
Part C: Needs related to ICC tasks of future jobs	Q7	Q7.1 – Q7.10 Closed-ended Q7.11 Open-ended	Tourism staff’s attitudes in intercultural communication with guests
	Q8	Q8.1 – Q8.10 Closed-ended Q8.11 Open-ended	The ICC real-world tasks in tourism industry

Specifically, the first part of the questionnaires provides the participants’ demographic characteristics. The second part explores the students’ ICC needs related to ETP courses which comprised the intercultural language learning activities suggested by Huber and Reynolds (2014) and adapted from Sercu (2005). The last part of the questionnaire are the real-world tasks related to ICC that the students need for their future jobs. This part was designed based on the framework for analyzing the factors contributing to tourism staff’s ICC (adapted from Byram, 2006; Yu et al., 2001) covering linguistic, sociolinguistic, discourse competences and intercultural competence. IC comprises affective, cognitive and behavioural capacities, i.e. intercultural attitudes, knowledge and behaviours. Hence, the first question in this section investigates the needs of ICC related to the attitudes that tourism staff should have when communicating with their guests (adapted from Inkaew, 2022). The second question in this part searches for the needs of the remained ICC dimensions in tourism field with real-world intercultural communicative tasks set up by Long’s (2005) task-based approach.

### Data Collection

A survey was employed to collect data from 628 undergraduates attending ETP courses at three educational institutions in central Vietnam. One junior college and two universities were chosen since ETP is one of the compulsory subjects to all of the tourism majored students in their final school year here; consequently, the teaching and learning English for this domain in those research sites was crucial. A description of this study’ purpose, an explanation of how the data would be collected and used as well as a link to the Google-form survey were sent to the administrators at these schools with their subsequent agreement and permission to conduct the data collection. Before participating in the survey, the participants read a statement explaining the questionnaire’s purpose and completed the consent form to have their data included in this study voluntarily.

### Data Analysis

#### Analyzing Questionnaire Data of Closed-ended Questions

Measures of central tendency (means and standard deviation) were used to analyze the data from the questionnaire. Mean scores (M) and standard deviation (S.D.) were employed as descriptive statistics calculated by SPSS (version 22). Mean scores of needs were interpreted as in Table 2.

**Table 2:** Interpretation of Mean Scores’ Range

Score range	Mean rating	Interpretation
4.21–5.00	Very important/Like most/Completely should have/Need most	Very high
3.41–4.20	Important/Like/Should have/Need	High
2.61–3.40	Neutral	Moderate
1.81–2.60	Not very important/Dislike somewhat/Should not have/Need somewhat	Low
1.00–1.80	Not important at all/Strongly dislike/Should not have at all/Do not need at all	Very low

#### Analyzing Questionnaire Data of Open-ended Questions

Thematic analysis (Braun & Clarke, 2006) were used for analyzing data from the open-ended parts of the questionnaires. This method of qualitative data analysis entails a process of reading again and again the whole data set to report repeated patterns, then identifying and refining themes (Braun & Clarke, 2006). Specifically, some initial categories were first created based on patterned key words and main ideas in the participants’ answers for open-ended questions. Then, these themes, after being analyzed and refined, used to code all the responses. A second rater who experienced in coding data used the given themes to code data independently.

### Reliability and Validity of the Study

Paltridge and Phakiti (2018, p. 31) explain that reliability is about “consistency in measuring something of interest. Often, reliability is associated with research instruments”. In this study, the instrument is the questionnaire which was tested with the Cronbach’s Alpha results as shown in Tables 3 and 4.

**Table 3:** Cronbach’s Alpha Reliability Statistics of The Undergraduates’ Questionnaire

Questionnaire	Cronbach’s Alpha	Number of items
ETP undergraduates’ responses	0.975	42

**Table 4:** Cronbach’s Alpha Reliability Statistics of Each Cluster in The Undergraduates’ Questionnaire

Clusters	Cronbach’s Alpha	Number of items
Favourite activities of learning ICC	0.954	7
Attitudes towards tourists in IC situations	0.881	10
ICC real-world tasks	0.964	10

To ensure the reliability of qualitative data analysis, a random sample of 20% of the participants’ responses was checked by a second rater. The inter-rater consistency ranged from 85 to 89% which is acceptable (Yin, 2011). The mismatch items were resolved through the raters’ discussion.

In addition, validity refers to the accuracy of research findings (Ho, 2011). In this study, satisfactory reliability and validity were achieved through careful consideration of research design, and the data was collected and analyzed explicitly.

## Findings

### Undergraduates’ ICC Needs Related to ETP Courses

The first five questions of the questionnaire collected the participants’ demographic information. In Question 6, the preferred intercultural learning activities were investigated thanks to seven intercultural language learning activities suggested by Huber and Reynolds (2014). First of all, from Table 5, it can be seen that undergraduates enjoyed all of given activities for learning ICC shown by high mean scores. Among these seven activities, speaking with a native English speaker had the highest mean score with M = 3.92, meanwhile the lowest mean score belonged to the task of talking about foreign countries’ literature with M = 3.58.

**Table 5:** Mean Scores of Undergraduates’ Choices of Preferred Intercultural Communicative Learning Activities (N = 628)

Items	ICLL activities	M	S.D.	Interpreted
1	Discussing with your friends or foreigners on social media what you heard (or read) about an English speaking country or culture.	3.88	1.07	High
2	Discussing with your friends why you find an image in the foreign culture(s) fascinating or strange.	3.74	1.07	High
3	Exploring an aspect of your own or foreign culture by films or texts in the Internet.	3.77	1.13	High
4	Communicating with a person originating from an English speaking country in your classroom.	3.92	1.07	High
5	Participating in role-play situations in which people from different cultures meet.	3.70	1.16	High
6	Comparing an aspect of your own culture with that aspect in an English speaking country’s culture.	3.67	1.15	High
7	Discussing actions or characters in an English speaking country’s literary work.	3.58	1.18	High
**Mean score**		**3.75**	**1.12**	**High**

Since all the open-ended questions in this study’s questionnaire were not compulsory for the participants to answer, there were only 396 responses which were classified in the following themes (Table 6). The most favoured activity to learn for ICC was communicating with various people in diverse situations with 261 participants’ comments. The second most frequent response was learning ICC through different cultural facets (58 responses). The students also preferred the activities of dealing with unexpected intercultural circumstances and integrating ICC with English language skills with two answers for each activity. Other intercultural language learning activities were also suggested such as learning ICC through verbal and non-verbal communication, experiential study, and well-known tourist spots. There was one mention of each activity.

**Table 6:** Summary of Undergraduates’ Responses to Add Intercultural Language Learning Activities (N = 396)

Themes	n	Representative response
Communicating with people from different cultures	261	“I prefer studying ICC by communicating with different people.”
Cultural issues	58	“I want to learn ICC through diverse cultural matters.”
Coping unexpected situations	22	“I like learning how to deal with unexpected intercultural situations.”
Integration ICC into four language skills	19	“My favourite activity is learning ICC and language skills concurrently.”
Verbal and non-verbal communication	12	“ICC can be improved by practising verbal and non-verbal language.”
Experiential learning	13	“I want to go to many places in my field trip.”
Tourist attractions	11	“We can learn intercultural knowledge through famous tourist spots.”

Note: Grammar in undergraduates’ responses was edited for clarity

### Undergraduates’ ICC Needs Related to Their Future Jobs

In this study’s questionnaire, Question 7 covers all the attitudes that the undergraduates thought tourism staff should or should not have in intercultural communication with guests. Meanwhile, Question 8 comprises all real-world tasks related to linguistic, sociolinguistic, discourse and intercultural competence (knowledge and behaviours) that tourism personnel need for their jobs.

#### Intercultural Communicative Language Learning Activities

Intercultural communicative language learning (ICLL) activities are designed to promote language learning while fostering intercultural understanding and communication skills. These activities encourage learners to explore cultural differences, develop empathy, and enhance their ability to effectively: Cultural Presentations, Cultural Comparisons, Language and Cultural Immersion Activities, Collaborative Projects, Cultural Reflection Journals etc. When designing ICLL activities, it is important to create a supportive and inclusive environment that encourages learners to share their perspectives and embrace cultural diversity. These activities not only enhance language proficiency but also foster empathy, respect, and intercultural competence, equipping learners with valuable skills for an increasingly interconnected world.

### Real-World Tasks

Real-world tasks in the context of language learning refer to activities that simulate or replicate authentic situations and tasks that learners may encounter outside the classroom. These tasks provide learners with opportunities to apply their language skills in practical and meaningful ways. Here are some examples of real-world tasks for language learning: role-play job interviews, problem-solving scenarios, group project presentations, group project presentations, etc. Real-world tasks offer learners the opportunity to apply their language skills in meaningful contexts, bridging the gap between the classroom and the outside world. By engaging in these tasks, learners develop practical language skills, critical thinking abilities, and the confidence to use their language competencies in real-life situations.

### Discourse and Behavioral Facets

Discourse and behavioral facets refer to specific aspects of communication and interaction that influence how individuals engage in conversations, express themselves, and interact with others. Here are some discourse and behavioral facets that can impact communication: turn-taking, non-verbal communication, empathy, clarity and conciseness, flexibility, constructive feedback, cultural sensitivity, conflict resolution etc. Developing and honing these discourse and behavioral facets can significantly enhance communication skills, build stronger relationships, and contribute to effective collaboration and understanding in various personal and professional context.

#### Perceptions of Attitudes That Tourism Staff Should or Should Not Have in Intercultural Communication with Guests

It can be seen from Table 7, among the group of attitudes with mean scores’ interpretation of “*very high*” and “*high*”, the attitudes of “*Having a positive view of people from different cultures*” and “*Treating every guest equally no matter what their religion or nationality*” had the highest mean scores, specifically M = 4.27 and M = 4.23 respectively. Next came the attitudes of “*Enjoying talking or working with foreign guests who are from different cultures*” and “*Enjoying working in an intercultural context*” (M = 4.08). “*Being curious and wanting to know about the cultural backgrounds of guests from other countries, looking for information in order to know how to treat them properly*” (M = 3.97) was placed at the last rank in this group.

**Table 7:** Mean Scores of Undergraduates’ Perceptions of Attitudes towards Tourists in Intercultural Communicative Situations (N = 628)

Items	Attitudes	M	S. D.	Interpreted
1	Having a positive view of people from different culture.	4.27	0.96	Very high
2	Being curious and wanting to know about the cultural backgrounds of guests from other countries, looking for information in order to know how to treat them properly.	3.97	1.09	High
3	Enjoying talking or working with foreign guests who are from different cultures.	4.08	1.03	High
4	Thinking all people are basically the same.	3.39	1.32	Moderate
5	Treating every guest equally no matter what their religion or nationality.	4.23	1.04	Very high
6	Feeling embarrassed when seeing people from other cultures exchanging greeting and goodbye kisses.	2.86	1.56	Moderate
7	Questioning the situation when seeing an old foreign tourist carrying things without any help from their children accompanying them.	3.11	1.43	Moderate
8	Sometimes feeling uncomfortable to provide help to some groups of foreign guests.	2.79	1.57	Moderate
9	Feeling nervous when having to service guests who are from a different culture.	2.99	1.46	Moderate
10	Enjoying working in an intercultural context.	4.08	1.04	High
**Mean score**		**3.58**	**1.25**	**High**

The remained attitudes interpreted as “*moderat*e” category were ranked in the following descending order of mean scores, i.e. “*Thinking all people are basically the same*” (M = 3.39), “*Questioning the situation when seeing an old foreign tourist carrying things without any help from their children accompanying them*” (M = 3.11), “*Feeling nervous when having to service guests who are from a different culture*” (M = 2.99), “Feeling embarrassed when seeing people from other cultures exchanging greeting and goodbye kisses” (M = 2.86), and lastly “*Sometimes feeling uncomfortable to provide help to some groups of foreign guests*” (M = 2.79).

Answering the question about other attitudes that tourism staff should have in intercultural communicative situations, 354 students had responses which listed other attitudes towards tourists such as being cheerful and sociable (146 responses), respectful of other cultures (83 responses), polite and friendly (57 responses), hospitable and enthusiastic (45 responses) as well as other attitudes. These included being patient, open-minded and truthful with one response for each attitude. Table 8 depicts the complete list of categories and example statements of the participants.

**Table 8:** Summary 0f Undergraduates’ Responses of Supplementary Intercultural Attitudes (N = 354)

Themes	n	Representative response
Cheerful and sociable	146	“Working in tourism industry, I must be cheerful and sociable”.
Respectful of tourists’ cultures	83	“I have to respect our guests’ cultures”.
Polite and friendly	57	“I should be polite and friendly with my clients”.
Hospitable and enthusiastic	45	“The first and foremost attitudes of labour in tourism field are hospitable and enthusiastic.”
Others	23	“Tourist staff need to be patient / open / honest.”

Note: Grammar in undergraduates’ responses was edited for clarity

Briefly, the findings of Question 7 revealed that the undergraduates believed the tourism professionals should have the positive attitudes such as enjoying talking or working with foreign guests, wanting to know about the guests’ cultural backgrounds in order to treat them properly, or having a positive view of people from different cultural backgrounds. Especially, they definitely should treat every guest equally no matter what their religion or nationality.

#### Perceptions of Intercultural Communicative Tasks Needed in Future Jobs

Overall students perceived that they needed all the given ICC tasks with the interpretation of all mean scores as “*high*” (Table 9). Particularly, the tasks related to discourse and behavioural competences, i.e. understanding customers’ verbal requirements thoroughly and handling unexpected circumstances properly, had the highest mean score (M = 3.94). On the contrary, the sociolinguistic task of having suitable small talk with different tourists held the lowest mean score (M = 3.78).

**Table 9:** Mean Scores of Undergraduates’ Perceptions of Intercultural Communicative Tasks Needed For Future Jobs (N = 628)

Items	Intercultural communicative tasks	M	S.D.	Interpreted
1	Giving guests instructions and advices about local customs	3.81	1.06	High
2	Providing services suitable to guests’ nationality cultures and characteristics	3.82	0.92	High
3	Demonstrating the ability of using English language (grammar, vocabulary, pronunciation) properly	3.87	1.05	High
4	Interpreting guests’ non-verbal enquiries well	3.85	1.07	High
5	Interpreting guests’ verbal enquiries well	3.94	1.05	High
6	Distinguishing the hosts - guests’ cultural differences	3.83	1.00	High
7	Initiating small talk with all guests appropriately	3.78	1.05	High
8	Dealing with unexpected situations with special groups of guests	3.94	0.98	High
9	Dealing with guests’ complaints related to different cultures	3.90	1.01	High
10	Developing rapport with tourists	3.90	1.09	High
**Mean score**		**3.86**	**1.03**	**High**

For other intercultural communication tasks (Table 10), there were 135 answers. The respondents believed that the tasks they needed in their future jobs included tasks related to intercultural knowledge (54 responses), behaviours (69 responses) and attitudes (12 responses). An example of intercultural cognitive task was exploring and understanding apparently the guests’ cultural background before they came in order to serve the guests well. Another illustration of behavioural task was dealing with requests of guests coming from different cultures. The participants also needed tasks pertaining to attitudinal facet such as being polite and flexible when dealing with guests’ faults. Interestingly, no intercultural tasks referring to linguistic, sociolinguistic and discourse competences were mentioned by the participants.

**Table 10:** Summary of Undergraduates’ Responses of Supplementary Intercultural Communicative Tasks (N = 135)

Themes	n	Representative response
Knowledge	54	“To prepare and provide the best service, I need to learn my clients’ culture even before their arrival.”
Behaviours	69	“Meeting multicultural customers’ needs is definitely necessary.”
Attitudes	12	“I need to be polite and deal with guests’ fault flexibly.”

Note: Grammar in undergraduates’ responses was edited for clarity

## Discussion and Implications

Generally, in this study, the undergraduates’ ICC needs included the learning needs analysis investigating the needs related to ETP courses, and the target needs analysis examining the ones associated with their future jobs (Long, 2005). Firstly, in the learning needs analysis, their ICC needs consisted of their favourite kinds of intercultural communicative language learning activities (Huber & Reynolds, 2014). Notably, the undergraduates’ preferred intercultural language activities included learning ICC by authentic intercultural communication situations, virtual educational tools or social network, and learning ICC combining with entertainment such as film extracts or text excerpts. Having combined both quantitative and qualitative data, it could be noticed that one of the learners’ favourite activities in learning ICC was communicating with people from diverse cultural background in various contexts, especially with native English speakers. These results match those observed in earlier studies. The students also preferred the pronunciation of teachers from English speaking country (Fuangkarn, K., & Rimkeeratikul, 2020) and authentic materials in their English classes in order to develop language competence and communicative skills (Smith et al., 2021). The source language and realia are considered as the basic foundation of teaching ESP (Basturkmen, 2010; Long, 2005). These results also offer compelling evidence for ETP educators to involve foreignness and authentic teaching materials in their intercultural language teaching. This could be done by various intercultural educational activities covering team-teaching with native-English-speaking teachers, apprenticeship abroad, exchange student programs, and intercultural-related seminars.

Secondly, in terms of target needs analysis, their needs identified by their attitudes towards customers and the necessary real-world tasks in workplaces (Inkaew, 2022; Long, 2005). In regard to the perceptions of attitudes that employees should have in intercultural communication with guests, the findings exposed that the undergraduates believed the tourism professionals *should not have* the attitudes such as feeling “embarrassed when seeing people from other cultures exchanging greeting and goodbye kisses”, or “questioning the situation when seeing an old foreign tourist carrying things without any help from their children accompanying them”. These findings coincided with those of Inkaew (2022) with the fact that the participants also did not approve these attitudes. Interestingly, there was one discrepancy between the two studies’ findings. On one hand, the undergraduates in Inkaew’ s (2022) study had low to moderate needs of the attitudes of enjoying “talking or working with foreign guests”, and “wanting to know about the guests’ cultural backgrounds in order to know how to treat them properly”. On the other hand, the student participants in this research believed that tourism staff *completely should have* these attitudes. A possible explanation for this might be that the participants in the current research were more open-minded and quickly accustomed themselves to the cultures of more and more foreign tourists coming to their home land due to the great internationalization in Vietnamese tourism industry. Hence, they were in favor of exploring foreign cultures as well as communicating and working in intercultural environment.

Additionally, the qualitative findings showed that, in conjunction with the mentioned attitudes in this study’ questionnaire, the undergraduates also noticed the other attitudes that tourism personnel should have such as cheerful and sociable (83 answers), respectful of other cultures (57 participants), polite and friendly (45 students), etc. The present findings are consistent with the affective competence of tourism staff suggested by Yu et al. (2001, p. 81) including being “*empathetic, non-judgmental and sensitive to others’ needs*”.

With reference to the needs related to real-world tasks in tourism professionals, the undergraduates showed their keen interests in all ICC dimensions, yet their least preference was the real-world tasks related to sociolinguistic competence. Surprisingly, the current study does not support previous researches with the fact that the task referring to sociolinguistic constituent was not considered as one of the undergraduates’ strong needs. It was documented that learners of tourism specialization needed most the sociolinguistic task of making daily life conversations (Rungsavang & Clarke, 2016). Nonetheless, in contradiction with earlier findings, students in this research perceived that the sociolinguistic task, i.e. greeting and initiating small talks with guests properly, was not as important as the other ICC tasks. This could be accounted for the participants’ strong emphasis on behavioural dimension of ICC tasks in tourism job duties such as meeting tourists’ requests, or dealing with guests’ complaints, showed by both quantitative and qualitative data, which may lead to their ignorance of the sociolinguistic tasks.

Remarkably, the findings revealed that one of students’ greatest needs related to behavioural competence. This result corroborates earlier findings when learners of tourism English also showed their intense interest of tasks belonging this competence such as supplying information, providing qualified services, and giving support (Prachanant, 2012).

Succinctly, in order to help students adapt to the intercultural communicative settings of tourism industry (Liu et al., 2022) and provide guests with high quality service (Prachanant, 2012), important implications for learners, educators and related stakeholders can be withdrawn. First of all, ETP students should raise their awareness of the fact that not only behavioural or a single intercultural dimension should be noticed, but other ICC components, for instance, sociolinguistic competence, should be focused simultaneously in learning ETP. This could be achieved by the fact that learners of ETP should learn to improve all ICC facets so that they are adequately qualified for their upcoming employment. Meanwhile, the teachers can incorporate all ICC dimensions’ practices into their English language classes regularly. In addition to intercultural teaching and learning, one of the issues emerging from this research’s findings is that professional managers may consider ICC as one of the prerequisites for hospitality and service personnel to better adapt to the international working environment with suitable attitudes and good performance of intercultural communicative tasks. These managers can help their employees to enhance this competence by training courses such as multinational training workshops or conferences, exchange employee programs or coaching related to intercultural communicative knowledge, behaviours and attitudes (Yang et al., 2020).

According to Corbett (2019), ESP in general and ETP in particularly focus on provide students with knowledge and skills to perform job duties in “*largely predictable and generalizable situations*”. However, language teaching and learning at higher level needs to improve learners’ capabilities and attitudes to undertake tasks in “*less predictable situations*” (p. 121). In order to achieve this goal, teaching and learning both English language proficiency and every single dimension of ICC should be emphasized concurrently in ETP classes.

## Conclusion

This study aimed to seek for learners’ needs of ICC related to their preference of intercultural language learning activities in ETP courses as well as their needs of necessary attitudes and real-life tasks when communicating with tourists in intercultural interactions. The findings provide more insights into the limited literature of NA pertaining to ICC particularly. The implications for tourism students, teachers and related parties are also presented. This study can be considered as a reference point for further studies investigating learners’ needs of ICC in tourism domain. The learners’ needs analysis and ICC have been advocated more attention from scholars; however, there are scant researches examining undergraduates’ needs of ICC, a vital competence of language learners in the era of globalization, especially in an Asian context. Hence, more and more studies investigating this issue will provide invaluable literature into this research topic.

## Limitations

This study has a few limitations. First, it could employ the participation of tourism majored students at a city in central Vietnam. Future research should be conducted with a larger sample from different regions and educational institutions in Vietnam to increase more generalization about needs of ICC in hospitality and tourism field. In addition, in this research, the findings were collected from the undergraduates merely. Further studies that investigate the needs analysis of ICC from related stakeholders such as teachers, graduates, professional experts would be noteworthy for both teaching and learning ICC in ELT generally and in ETP particularly. Despite these limitations, the study has offered a useful insight of tourism majored undergraduates’ needs analysis of ICC at tertiary level in Vietnamese context.
